# Inefficient N2-Like Neutrophils Are Promoted by Androgens During Infection

**DOI:** 10.3389/fimmu.2018.01980

**Published:** 2018-09-03

**Authors:** María V. Scalerandi, Nahuel Peinetti, Carolina Leimgruber, Mariana M. Cuello Rubio, Juan P. Nicola, Gustavo B. Menezes, Cristina A. Maldonado, Amado A. Quintar

**Affiliations:** ^1^Centro de Microscopía Electrónica, Facultad de Ciencias Médicas, Universidad Nacional de Córdoba, Córdoba, Argentina; ^2^Instituto de Investigaciones en Ciencias de la Salud (INICSA), Consejo Nacional de Investigaciones Científicas y Técnicas (CONICET), Córdoba, Argentina; ^3^Departamento de Bioquímica Clínica, Facultad de Ciencias Químicas, Universidad Nacional de Córdoba, Córdoba, Argentina; ^4^Centro de Investigaciones en Bioquímica Clínica e Inmunología (CIBICI), Consejo Nacional de Investigaciones Científicas y Técnicas (CONICET), Córdoba, Argentina; ^5^Center for Gastrointestinal Biology, Instituto de Ciências Biológicas, Universidade Federal de Minas Gerais, Belo Horizonte, Brazil

**Keywords:** neutrophils, testosterone, androgens, bacterial prostatitis, infection

## Abstract

Neutrophils are major effectors of acute inflammation against infection and tissue damage, with ability to adapt their phenotype according to the microenvironment. Although sex hormones regulate adaptive immune cells, which explains sex differences in immunity and infection, little information is available about the effects of androgens on neutrophils. We therefore aimed to examine neutrophil recruitment and plasticity in androgen–dependent and –independent sites under androgen manipulation. By using a bacterial model of prostate inflammation, we showed that neutrophil recruitment was higher in testosterone-treated rats, with neutrophil accumulation being positively correlated to serum levels of testosterone and associated to stronger inflammatory signs and tissue damage. Testosterone also promoted LPS-induced neutrophil recruitment to the prostate, peritoneum, and liver sinusoids, as revealed by histopathology, flow cytometry, and intravital microscopy. Strikingly, neutrophils in presence of testosterone exhibited an impaired bactericidal ability and a reduced myeloperoxidase activity. This inefficient cellular profile was accompanied by high expression of the anti-inflammatory cytokines IL10 and TGFβ1, which is compatible with the “N2-like” neutrophil phenotype previously reported in the tumor microenvironment. These data reveal an intriguing role for testosterone promoting inefficient, anti-inflammatory neutrophils that prolong bacterial inflammation, generating a pathogenic environment for several conditions. However, these immunomodulatory properties might be beneficially exploited in autoimmune and other non-bacterial diseases.

## Introduction

Neutrophil granulocytes lead the initial leukocyte influx to sites of injury in order to eliminate invading pathogens or damaged tissues. Their response is mediated by phagocytosis and NETosis, as well as by releasing defensins, enzymes, and cytokines to active the immune response (1). Subsequently, once the inflammatory stimuli has been eradicated, neutrophils die by apoptosis and the elimination of apoptotic bodies by macrophages ensures the correct resolution of the inflammatory process and tissue repair (2). However, if the inflammatory process is not controlled, the products generated by neutrophils can induce multiple tissue alterations and loss of cellular function (3, 4). This is particularly important in endotoxemia- and burn-induced multiple organ dysfunction and in unresolved infections of the reproductive tract, where neutrophil activation could be harmful causing degradation of the extracellular matrix and additional gamete damage beyond that associated with the initial injury (5–7).

Although for decades it was thought that neutrophils constitute a homogeneous cell population, reports of neutrophils showing different behaviors in front of diverse steady state and pathological conditions are shifting this notion (8–10). For instance, neutrophils are able to shape and regulate immune and inflammatory responses against tumor cells by exerting either pro-tumor or anti-tumor effects on tumor development (9, 11). Given these varied effects, the concept of neutrophil plasticity and diversity has emerged, leading to the paradigm of anti-tumoral “N1 neutrophils” vs. pro-tumoral “N2 neutrophils” proposed by Fridlender and coworkers (9), in neoplastic scenarios. However, little is known about different neutrophil phenotypes promoted by non-tumoral environments as metabolic or hormonal imbalance.

The prostate gland is the main target of infectious and inflammatory diseases in the male reproductive tract, with prostatic inflammation representing a worldwide health issue. Moreover, a strong relationship between prostatitis and other conditions with high impact on human health such as male infertility (12), benign prostatic hyperplasia (13), and prostate cancer (14) has been reported. Unlike many organs in the body, the prostate is under the strict control of testicular male hormones for its development and function. Hence, it is not surprising that testosterone might influence the expression of pro- and anti-inflammatory mechanisms, as well as the local production of cytokines and chemokines, the recruitment and activation of immune cells, and the outcome of infectious diseases of the prostate gland. In this sense, we previously reported that testosterone negatively modulates the Toll-like receptor 4 (TLR4) pathway and downregulates antimicrobial substances in prostatic cells, which correlates with a decreased inhibition of bacterial growth in the presence of testosterone in both *in vivo* and *in vitro* models (15).

It has long been recognized that androgens dampen host defenses through multiple mechanisms of the adaptive immunity, explaining the sex-specific biases reported in immunocompetence, autoimmunity, and cancer incidence (16). Androgenic effects include apoptosis of T and B cells and the induction of T regulatory cells and CD8+ suppressive cells (16, 17). Additionally, in monocytes/macrophages, androgens reduce proinflammatory signaling (TLR4, TNFα, IL1β, and IL6) (18) but enhance anti-inflammatory (IL10) cytokine production (19). Regarding innate immunity, a few published articles have suggested that testosterone maintains a reduced expression of key elements such as TLRs and modulates the activity of different professional cells (18, 20). Nevertheless, the influence of androgen levels on neutrophil activity and plasticity in the initial inflammatory response remains to be investigated. Therefore, the aim of this work was to determine whether testosterone is able to modulate neutrophil recruitment and behavior in androgen dependent- and independent-sites.

## Materials and methods

### Animals

Wistar strain male rats, 12 weeks old, and weighing 250–350 g, were housed at the Animal Research Facility of the Universidad Nacional de Cordoba, in air-conditioned quarters, under a controlled photoperiod (14 h light/10 h darkness) with free access to rodent food and tap water. C57BL/6 mice were from Centro de Bioterismo in Universidade Federal de Minas Gerais (CEBIO, UFMG, Brazil). Animal care and experiments were conducted following the recommendations of the International Guiding Principles for Biomedical Research Involving Animals and approved by the local CICUAL (FCM-UNC, Argentina) Ethical Committee.

### Androgen manipulation and prostatitis models

Rats were orchidectomized via the scrotal route under ketamine (80 mg/kg)/ xylazine (8 mg/kg) and divided into three groups, receiving immediately testosterone s.c. at physiological (2 mg/kg/day; T group; Sustanon, Organon, Argentina) or supraphysiological doses (10 mg/kg/day; TT group), or vehicle alone (sunflower oil; OX group). To confirm the androgen status, serum total testosterone levels of individual rats were determined by electrochemiluminescence immunoassay using a Roche Elecsys E170 immunoassay analyzer (Roche Diagnostics GmbH, Mannheim, Germany).

An acute bacterial prostatitis model was performed according to a protocol from our laboratory (21, 22). Briefly, two days after castration, OX, T, and TT rats were anesthetized and subjected to a laparotomy to expose the ventral prostate; infection was induced by an intraprostatic injection of 200 μL of *Uropathogenic E. coli* (10^8^ CFU/ml, isolated from a patient with complicated urinary tract infection), with a 30-gauge needle. Animals were killed at 1, 3, and 5 days after infection, with the ventral prostate being processed for morphological, biochemical, and microbiological analyses. As controls, rats subjected to the same surgical procedures were used, replacing the bacterial suspension with sterile PBS.

On the other hand, a lipopolysaccharide (LPS)-induced model for prostatic inflammation was carried out by inoculating 50 μl of a solution of LPS from *E. coli* 055:B5 (20 mg/ml, Sigma–Aldrich, St. Louis, MO) in OX and T groups using the same surgical procedure described for bacterial prostatitis. Control animals received the vehicles. Ventral prostates were harvested and processed at 24 h after inoculation.

### Neutrophil recruitment to the peritoneum and microbial killing assay

Neutrophil recruitment was induced by a single i.p. injection of thioglycollate (3 ml of a 3% solution; Sigma–Aldrich, St. Louis, MO) or LPS from *E. coli* 055:B5 (1 mg/kg) in rats of OX and T groups at day 3 post-castration. Sterile saline-injected animals were used as controls. Animals were anesthetized 4 h after thioglycollate or 12 h after LPS injection and the peritoneal lavage was harvested for analysis by injection of 10 mL of sterile PBS containing 0.835 UI/mL sodium heparin. The abdomen was gently massaged and the blood-free cell suspension was carefully aspirated with a syringe. The cell suspension was then spun for 6 min, 300 × g, at 20°C. After removal of the supernatant, residual red blood cells were removed by hypotonic lysis and the cells were spun again for 6 min, 300 × g, at 20°C. The cell pellet was washed and resuspended in 2 ml HBSS and total leukocyte counts were performed immediately in peritoneal lavage samples using a Neubauer chamber. To evaluate the neutrophil percentage, the differential leukocyte population was analyzed in cytospins after May-Grünwald-Giemsa staining (Biopur, Rosario, Argentina); a minimum of 500 leukocytes were counted, containing >85% pure populations of neutrophils. Additionally, peritoneal cells were stained with a FITC anti-rat granulocyte (Gr) antibody that recognizes rat neutrophils (BD Biosciences, San Jose, CA) and analyzed by flow cytometry using a FACSCanto II cytometer (Becton Dickinson, San Jose, CA).

For assessing *ex vivo* neutrophil bactericidal activity, thioglycollate-recruited neutrophils from OX and T groups were counted manually using a standard hemocytometer. Cytospins confirmed >90% neutrophils. *Uropathogenic E. coli* (the same used for prostatitis model) were pre-opsonized in 10% mouse serum on ice for 15 min. Neutrophils in RPMI 1640 (Sigma–Aldrich, St. Louis, MO) were plated onto 24 well plates at 1 × 10^6^ neutrophils/well and infected with equal volume of *E. coli* in serum at a multiplicity of infection of 1 bacterium:1 neutrophil. Following 10 and 40 min incubation times, 50 μl of the suspension were taken for bacterial colony-forming units (CFU) quantification by serial agar plating.

### Intravital liver imaging

Mice were treated with testosterone (i.p. 10 mg/kg/day) or flutamide (s.c 7 mg/kg/day; Sigma–Aldrich, St. Louis, MO) for 3 days. Then, neutrophil recruitment was induced by a single i.p. injection of LPS from *E. coli* O111:B4 (0.5 mg/kg, Sigma–Aldrich, St. Louis, MO). Six hours after, mice received a single i.v. dose of a FITC anti-mouse Ly-6G antibody (4 μg/mouse; BioLegend, San Diego, CA), diluted in sterile saline (in a total volume of 100 μl), and confocal intravital imaging was performed as described (23, 24). In brief, mice were anesthetized (i.p.) with a mixture of ketamine (60 mg/kg) and xylazine (15 mg/kg) and a midline laparotomy was performed to expose the liver for imaging. Mice were imaged using Nikon Eclipse Ti (Nikon, Tokyo, Japan) with a C2 confocal head equipped with three different lasers (excitation at three wavelengths: 405, 488, and 543 nm) and emission bandpass filters at 450/50, 515/30, and 584/50 nm. The z-position is controlled by an automated device and 10X objective was used on the required resolution. Ten-minute movies were taken from each mouse and Ly-6G (+) neutrophil quantification was performed using Volocity 6.3 software (PerkinElmer, Waltham, MA).

### Histopathological analysis and immunocytochemistry

Tissue samples of ventral prostates were formalin-fixed and paraffin-embedded for routine hematoxylin–eosin (H&E) staining (Biopur, Rosario, Argentina). An Axiostar Plus microscope equipped with a digital camera (Zeiss, Oberkochen, Germany) was used to acquire 60X photographs, which were examined using Fiji software (NIH, Bethesda, MD). For prostate neutrophil quantification, 20 fields of 2 different sections from the same gland were analyzed, with at least three animals per experimental group.

Immunocytochemistry was performed on slides from paraffin-embedded prostates, which were cleared with xylene and rehydrated in a descending concentration series of ethanol. Samples were then incubated in EDTA pH 9.0 to perform antigen retrieval using microwave pre-treatment (except for detection of *E. coli*). To block the endogenous peroxidase activity, slides were treated with H_2_O_2_ in methanol for 15 min. Sections were treated with PBS-BSA 5% to block non-specific binding for 30 min, followed by an overnight incubation with the primary antibody (diluted in 1% PBS-BSA) at 4°C in a humidified chamber. Afterwards, slides were incubated for 1 h with a specific biotinylated secondary antibody (at 1:180; Amersham Pharmacia, Buckinghamshire, UK) followed by 30 min in ABC complex (Vector, Burlingame, CA). Diaminobenzidine (Sigma–Aldrich, St. Louis, MO) was used as a chromogen substrate for 10 min and Harris hematoxylin as a counterstaining solution. Primary antibodies used for this study were: anti-PBP (rabbit polyclonal at 1:2000, developed by Dr. Maccioni (22), anti-ACTA2 (mouse monoclonal at 1:50; Novocastra, Newcastle, UK), and anti-*E. coli* antigen (rabbit polyclonal at 1:250, Affinity BioReagents, Golden, CO). For negative controls, antibodies were pre-absorbed with specific blocking peptides or replaced by rabbit or mouse normal serum.

### Transmission electron microscopy

Tissue blocks (1 mm^3^) from ventral prostate and pellets of peritoneal neutrophils were fixed in Karnovsky's mixture containing 2% (v/v) glutaraldehyde (EMS, Hatfield, PA) and 4% (w/v) formaldehyde in 0.1 M cacodylate buffer, pH 7.3, at 4°C for 24 h. Samples were post-fixed in 1% osmium tetroxide (EMS, Hatfield, PA) for 2 h and washed in 0.1 M cacodylate buffer, before being dehydrated through a graded series of cold acetone and embedded in Araldite epoxy resin (EMS, Hatfield, PA) as previously published (21, 22, 25). Ultrathin sections were cut in a JUM-7 ultramicrotome (Jeol, Tokyo, Japan) and examined in a Zeiss LEO 906E electron microscope (Zeiss, Oberkochen, Germany) with digital acquisition of images.

### Sorting of prostate infiltrating neutrophils by flow cytometry

Ventral prostates from rats with LPS-induced prostatitis were quickly excised, rinsed in fresh saline and weighted. Cell dissociation and neutrophil purification were performed using an adaptation of a published protocol (26). Briefly, tissues were minced into small fragments and treated with a digestion solution containing 200 U/ml collagenase type IA (Sigma Aldrich, St. Louis, MO) and 0.05% deoxyribonuclease type I (Sigma Aldrich, St. Louis, MO) in HBSS without calcium, magnesium or phenol red, pH 7.4 for 30 min, at 37°C, and rocking at 60 rpm. The digested tissue was then passed through a 70-μm pore cell strainer with fresh sterile PBS. The cell suspension was then spun for 6 min, 300 × g at 20°C. After removal of the supernatant, residual red blood cells were removed by hypotonic lysis and cells were spun again for 6 min, 300 × g, at 20°C, washed, and resuspended in 1 ml of PBS. Cells were counted by collecting events for a fixed time (90 s) on a FACSCanto II cytometer. Neutrophil percentage was calculated by using a FITC anti-rat Gr monoclonal antibody (1/500 for 1 h at 4°C).

To purify prostate-infiltrating neutrophils, Gr (+) cells were sorted in a FACSAria II cell sorter (BD Biosciences, San Jose, CA), with a purity of >97% being achieved and confirmed by electron microscopy.

### Measurement of myeloperoxidase (MPO) activity

Whole ventral prostate glands were removed, frozen in liquid nitrogen, and stored at −80°C. After thawing, tissue was weighed and homogenized on ice in 50 mM potassium phosphate buffer (1 g in 10 ml, pH 6.0). Homogenates were centrifuged (30.000 × g, 15 min, 4°C) and pellets were resuspended in hexadecyltrimethylammonium bromide (HTAB; Sigma Aldrich, St. Louis, MO) buffer (0.5% HTAB in 50 mM phosphate buffer, pH 6.0). Lysates were sonicated twice for 10 s each and freeze-thawed three times, after which sonication was repeated. Suspensions were then centrifuged (20.000×g, 15 min, 4°C) and the resulting supernatants were assayed for MPO activity. Supernatants (30 μl) were added to 970 μl of 50 mM phosphate buffer (pH 6.0) containing 0.167 mg/ml o-dianisidine hydrochloride (Sigma Aldrich, St. Louis, MO) and 0.0005% H_2_O_2_ and the change in absorbance at 460 nm (A460) was measured. One unit (U) of MPO activity was defined as that degrading 1 μmol of H_2_O_2_ per minute at 25°C and results were expressed as U MPO activity/g prostate and U MPO activity/mg protein, determined by the Bio-Rad Protein Assay kit (Bio-Rad Laboratories, Hercules, CA).

To assess MPO activity in peritoneal cells, cellular MPO was extracted with 0.5% HTAB buffer from 4 × 10^6^ cells obtained after hypotonic lysis of residual red blood cells. Lysates were then sonicated twice for 10 s each and spun for 15 min, 40.000×g, at 4°C. Resulting supernatant was assayed for MPO activity as described, with results being expressed as U MPO activity/mg protein and μU MPO activity/cells.

### Microbiological studies

To evaluate *in vivo* the growth of *E. coli*, pieces of ventral prostate from each rat were weighed, minced and gently homogenized in tryptic soy broth (1 g of tissue in 20 ml) in sterile conditions. Then, serial dilutions, i.e., 1/5 to 1/50, were made and 100 μl of these solutions were spread on Mueller Hinton agar, with the plates being incubated overnight at 37°C. Finally, bacterial counting was expressed as CFU per mg of prostatic tissue.

### *Ex vivo* determination of IL10 production

Wistar rats were treated with flutamide (s.c 7.5 mg/kg/day) or its vehicle for 5 days. Peritoneal cells were harvested after 4 h of thyoglycollate i.p injection, washed, and resuspended in HBSS as described above. Total cells were then plated onto 24 well plates (1 × 10^6^ neutrophils/well in RPMI 1640) and pulsed *ex vivo* with LPS O55:B5 1 μg/ml for 24 h to elicit cytokine secretion. *In vitro* assays maintained the same conditions as *in vivo* (i.e., flutamide stimulation). Immunofluorescence for IL10 in neutrophils was carried out on methanol-fixed, permeabilized cells on coverslides using PE anti-rat IL10 (1/50; BD Biosciences, San Jose, CA) and FITC anti-rat Gr (1/50) antibodies.

For flow cytometry, cells were treated with cycloheximide 100 μM (Sigma Aldrich, St. Louis, MO) 3 h before the analysis. The cell concentration was adjusted to 5 × 10^5^ cells/ml and labeled for 30 min with the following antibodies: PE anti-rat IL10 (1/60), APC anti-rat CD11b (1/100; BD Biosciences, San Jose, CA), and FITC anti-rat Gr (1/150). Signals were acquired in a FACSCanto II cytometer and analyzed using FlowJo X software (Tree Star, Ashland, OR).

### Immunoblotting

Prostate tissues were minced and homogenized on ice with a teflon-glass tissue grinder in 2 ml cold PBS containing 1.25% Igepal CA-630, 1 mM EDTA, 2 mM PMSF, 10 ug/ml leupeptin, and 10 ug/ml aprotinin. The lysate was centrifuged at 14,000 × g for 20 min at 4°C and the supernatant was withdrawn and stored in aliquots frozen at −70°C until required. Prostatic lysates from triplicate experimental conditions were pooled before loading into electrophoresis gels. Total protein concentration was measured with a Bio-Rad Protein Assay kit. For western blot, denatured protein samples (45 μg/lane) were separated on 12% SDS polyacrylamide gel and blotted to a Hybond-C membrane (Amersham Pharmacia, Freiburg, Germany). Incubation steps were performed in 5% defatted dry milk in PBS/0.1% Tween 20. Blots were incubated for the detection of SP-D (rabbit polyclonal antibody at 1:1000; Chemicon, Temecula, CA) during 3 h. After that, membranes were treated with peroxidase-conjugated goat anti-rabbit antibodies (Jackson, West Grove, PA) and visualized applying the chemiluminescence technique. The expression of β-actin (mouse monoclonal antibody at 1:5000, Sigma Aldrich, St. Louis, MO) was used as an internal control to confirm the equivalent total protein loading.

The peptide βdefensin-1 (HBD-1) was tested in homogenates of ventral prostates by dot blot. For that purpose, prostatic lysates were matched at a concentration of 100 μg/ml, and 4 μl of each sample was spotted onto a Hybond C Super membrane (Amersham-Pharmacia, Freiburg, Germany). The membrane was then treated as explained above for western blot using an anti-HBD-1 (at 1:250, Santa Cruz Biotech, Santa Cruz, CA) as primary antibody.

Semiquantitative signals were derived by densitometric analysis from western and dot blots using Fiji software and data were displayed as area units.

### ELISA

In order to quantify TNFα in prostate homogenates, tissues were minced and homogenized on ice in cold PBS, as described for immunoblotting, centrifuged at 4°C, 1.400 rpm for 15 min and stored at −20°C until the day of the assay. TNFα amount was measured by a commercially available sandwich ELISA kit (eBioscience, San Diego, CA), following the manufacturer's instructions.

### Measurement of gene expression by quantitative real-time polymerase chain reaction (qPCR)

Total RNA samples were extracted from peritoneal neutrophils and prostate Gr (+)- and Gr (–)-sorted cells using TRIzol (Thermo Fisher, Carlsbad, CA). RNA was subsequently purified using Direct-zol RNA miniprep kit (Zymo Res., Irvine, CA) according to manufacturer's instructions. Then, 0.5 μg of RNA was used as a template for reverse transcription using EpiScript™ Reverse Transcriptase (Epicentre, Madison, WI) with random hexamer primers (Fermentas, Thermo Fisher, Carlsbad, CA). qPCR was performed with power SYBR green PCR master mix (Applied Biosystems, Foster City, CA) in a ABI Prism 7500 detection system (Thermo Fisher, Carlsbad, CA). The expression of ACTB was chosen as housekeeping gene. Data analysis was based on the 2^−ΔΔ*Ct*^ method for normalization of raw data. All primers used are described in the Supplementary Table 1.

### Statistical analysis

The characterization of data was accomplished by comparing their mean values ± standard error of the mean (SEM) from at least four independent protocols. Statistical differences between means were analyzed by unpaired Student's *t*-test. Data from more than two groups were analyzed using analysis of variance (ANOVA) with Tukey as the post-test to compare all pairs of columns. Significant differences were considered at *p* < 0.05. Statistical analyses and graphics were performed using SPSS, version 23.0 (SPSS Inc., Chicago, IL) and GraphPad Prism 6 (La Jolla, CA).

## Results

### Androgen withdrawal modifies the progression of the inflammatory response in the prostate gland

**Figure 1:**
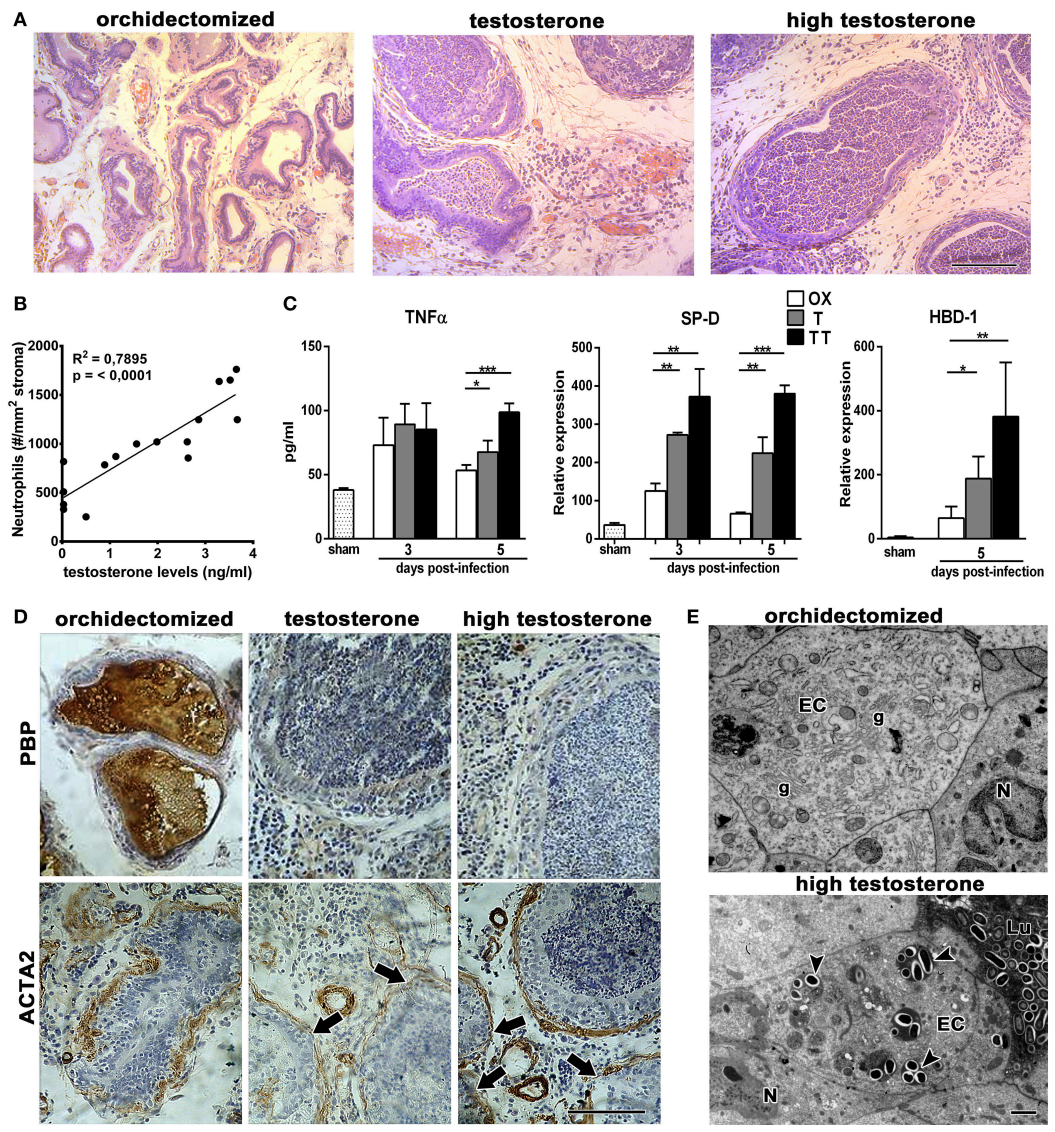
Androgens increase neutrophil infiltration and tissue damage in bacterial infection of the prostate gland. Rats were orchidectomized (OX) and treated with testosterone 2 mg/kg/day (T) or 10 mg/kg/day (high testosterone-TT) before being inoculated with *E. coli* intraprostatically. **(A)** H&E staining at day 5 after infection shows not only interstitial inflammatory infiltrates but also a massive neutrophil invasion to prostatic acini in testosterone-treated animals. Bar = 100 μm. **(B)** Correlative analysis showing serum testosterone levels and prostatic neutrophil counts (*n* = 16, Pearson's correlation test). Neutrophils counts were calculated on H&E sections at day 5 post-infection. **(C)** The inflammatory parameters TNFα, SP-D, and HBD-1 are strongly increased in the prostate from testosterone-treated animals, with the TT group showing the highest levels (mean ± SEM; *n* = 4 per group; *p < 0.05; **p < 0.01; ***p < 0.001). **(D)** Representative images of immunocytochemistry for PBP and ACTA2, at day 5 post-infection, displaying a loss in epithelial secretory function when testosterone levels remain high (top images). ACTA2, as a marker for stromal organization, shows a weak expression and disruptions in the periacinar layer (arrows in bottom) of these animals. Bar = 100 μm. **(E)** At ultrastructural level, prostatic damage was related to the presence of numerous bacteria in the lumen (Lu) and invading epithelial cells (arrowheads, bottom). Orchidectomized rats show conserved morphology, with the conservation of secretory organelles such as Golgi complexes (g). EC, epithelial cell; N, neutrophil. Bar = 2 μm.

In order to study the effects of testosterone on prostatitis progression, castrated animals treated with different doses of testosterone were subjected to intraprostatic bacterial inoculation and analyzed at days 1, 3, and 5 after infection. As shown in Figure 1A, the prostate from rats supplemented with testosterone exhibited a massive neutrophil infiltration and invasion into the prostatic acini; this effect was more evident at day 5 post-infection. Moreover, serum androgen levels positively correlated to the number of neutrophils infiltrating the gland (Figure 1B). In contrast, rats with low testosterone levels displayed reduced inflammatory signs, with few focal infiltrates in the prostatic interstitium. Accordingly, the prostatic levels of TNF-α, SP-D, and HBD-1 were higher in rats treated with high doses of testosterone (Figure 1C), demonstrating a stronger inflammatory reaction in these animals. Considering the deleterious effects of neutrophils on tissue function, we assessed the histoarchitecture as well as the expression of prostatic binding protein (PBP) and α-smooth muscle actin (ACTA2, markers of epithelial secretory function and stromal integrity respectively) of the prostate gland. At day 5 post-infection, both the secretory and the stromal compartments of the ventral prostate were conserved in the castrated animals as judged by PBP and ACTA2 expressions (Figure 1D). This was consistent with the conservation of ultrastructural features, including a prominent Golgi complex, developed endoplasmic reticulum, and secretory vesicles (Figure 1E). Conversely, the epithelial layer of the testosterone-treated rats exhibited atrophy, desquamation, and invasion of neutrophils, suggesting that the exacerbated presence of neutrophils is associated to more inflammatory signs and tissue damage in the prostate gland of animals with high levels of testosterone. Taking together, these results evidence adverse effects of testosterone in a dose dependent manner during bacterial acute prostatitis.

### Testosterone increases neutrophil recruitment independently of the stimulus nature or the site of injury

**Figure 2:**
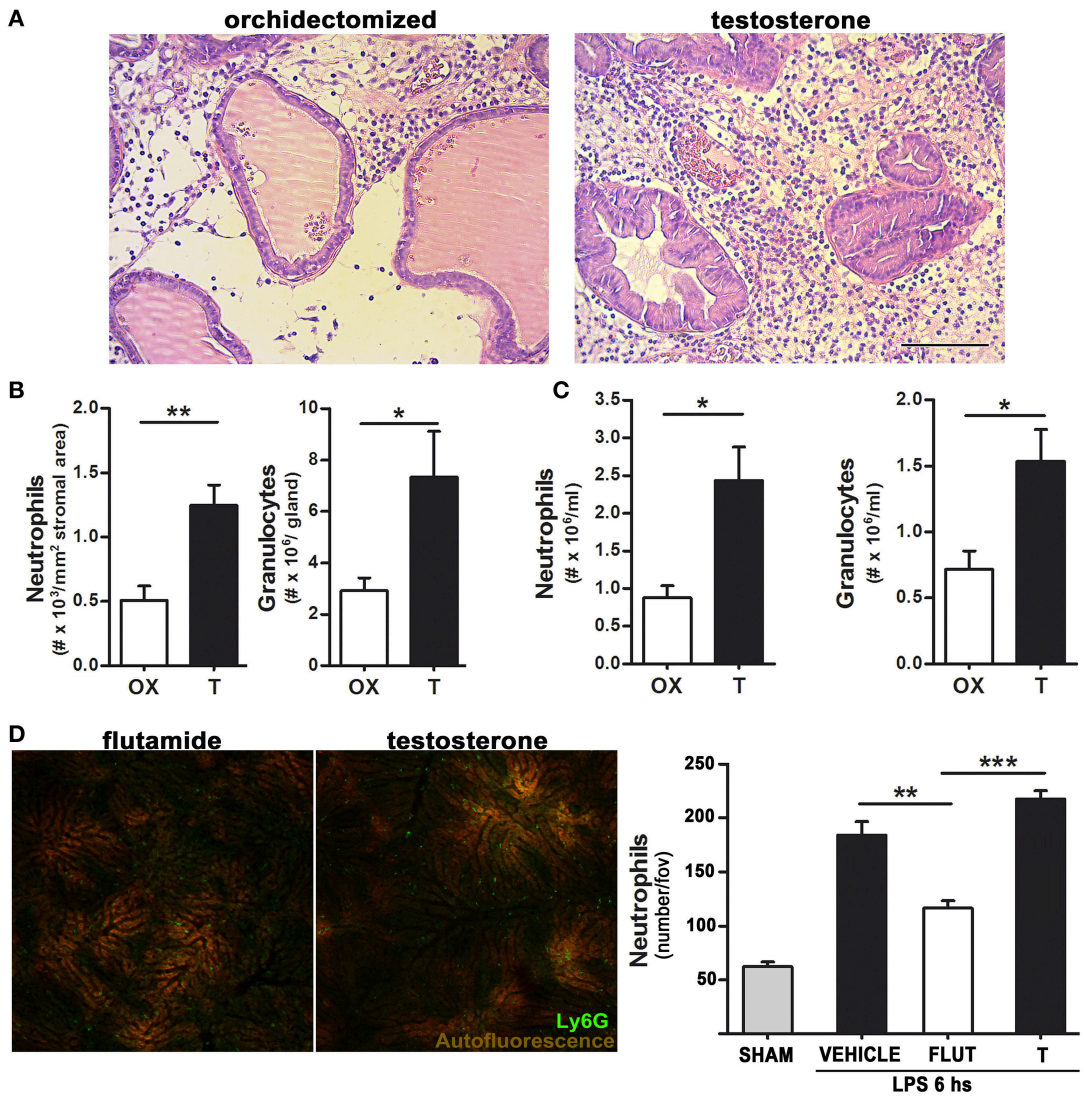
Testosterone signaling favors LPS-induced neutrophil recruitment in both androgen-dependent and –independent sites.**(A)**–**(B)** Rats were orchidectomized (OX) and treated with testosterone 2 mg/kg/day (T) before being inoculated with 1 mg of LPS intraprostatically for 24 h. **(A)** Representative H&E staining showing an intense neutrophil infiltration in testosterone-treated animals. Bar = 100 μm. **(B)** Quantification of neutrophil recruitment to the prostate in H&E-stained slides (left) and by flow cytometry using a FITC anti-Gr antibody (right). **(C)** Peritoneal neutrophil recruitment was achieved by injecting LPS 1 mg/kg for 12 h, with the quantification of neutrophils being performed by hemocytometer (left) and flow cytometry (right). **(D)** Neutrophil recruitment in the liver observed by intravital microscopy after an i.p. LPS 0.5 mg/kg injection. Mice were previously treated with testosterone (T; 10 mg/kg/day) or with the inhibitor of androgen signaling flutamide (FLUT; 7 mg/kg/day). The presence of testosterone increases neutrophil recruitment to the liver sinosoids. Left: representative images (see Supplementary Movie 1). Right: quantification of Ly6G (+) neutrophils per field of view. Data represent the mean ± SEM from at least three independent animals. *p < 0.05; **p < 0.01; ***p < 0.001.

The higher amount of neutrophils observed in testosterone-treated animals could be attributed to an androgen-related neutrophil malfunction which, unable to eradicate bacteria, maintains a constant recruitment of inflammatory cells. In order to further understand this, we used bacterial LPS instead of the live bacterium as stimulus. In line with the bacterial-induced prostatitis model, LPS promoted an intense inflammatory infiltration in testosterone-treated animals, resulting in a higher number of neutrophils within the prostate, assessed in histological slides as well as by flow cytometry using a specific antibody (anti-Gr) for rat neutrophils (Figures 2A,B).

Testosterone-treated rats also displayed a higher thioglycollate-induced neutrophil recruitment to the peritoneum (not shown), with this effect being even stronger when neutrophil recruitment was elicited by LPS for 12 h (Figure 2C), indicating that testosterone is associated to a higher recruitment of neutrophils not only in androgen-dependent but also in androgen-independent sites. Indeed, to corroborate this notion, a widely validated intravital mouse model (23, 24, 27) for visualization of LPS-induced neutrophil recruitment to the liver was performed. In mice treated with testosterone, LPS injection caused a massive neutrophil adhesion to hepatic sinusoids, which was reduced by the testosterone inhibitor flutamide (Figure 2D and Supplementary Movie 1).

**Figure 3:**
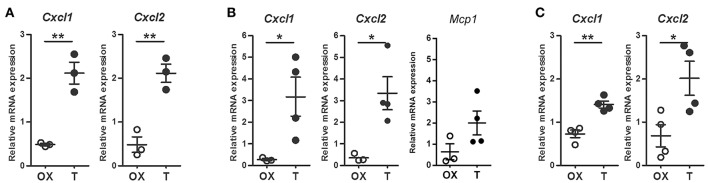
Androgens augment LPS-induced, neutrophil-specific chemokine mRNA expression. **(A**–**B)** Rats were orchidectomized (OX) and treated with testosterone 2 mg/kg/day (T) before being inoculated with 1 mg of LPS intraprostatically for 24 h. Cell sorting was carried out to isolate Gr (+) neutrophils from prostatic cells and mRNA expression was evaluated by qPCR. **(A)** Testosterone treatment promotes an increase in CXCL1 and CXCL2 in prostatic cells. **(B)** Neutrophils exhibit a similar behavior, with no changes in Monocyte Chemoattractant Protein-1 (MCP1), the key chemokine regulating migration of monocytes/macrophages. **(C)** LPS-induced peritoneal neutrophils from testosterone-treated animals (T) showing an increase in CXCL1 and CXCL2. In all cases, the mRNA levels are relative to those of ACTB. Mean ± SEM, each dot represents one animal. *p < 0.05; **p < 0.01.

Taking in mind that neutrophil recruitment from peripheral blood is a consequence of local chemokine production, mainly CXCL1 and CXCL2 (28), their mRNA expressions were analyzed in cells from prostates and peritoneal cavity after LPS challenge. As shown in Figure 3A, both chemokines increased after testosterone restoration in resident prostatic cells. Moreover, CXCL1 and CXCL2 were enhanced in Gr(+)-sorted neutrophils infiltrating the gland in the LPS-induced prostatitis model (Figure 3B) as well as in LPS-elicited peritoneal cells from animals treated with testosterone (Figure 3C). Interestingly, the expression levels of MCP-1 did not change significantly between groups (Figure 3B). Altogether, these data indicate that androgens promote neutrophil recruitment in both androgen-dependent and –independent sites by increasing the expression of specific chemokines in local cells as well as in neutrophils.

### Androgens decrease the bactericidal ability of neutrophils

**Figure 4:**
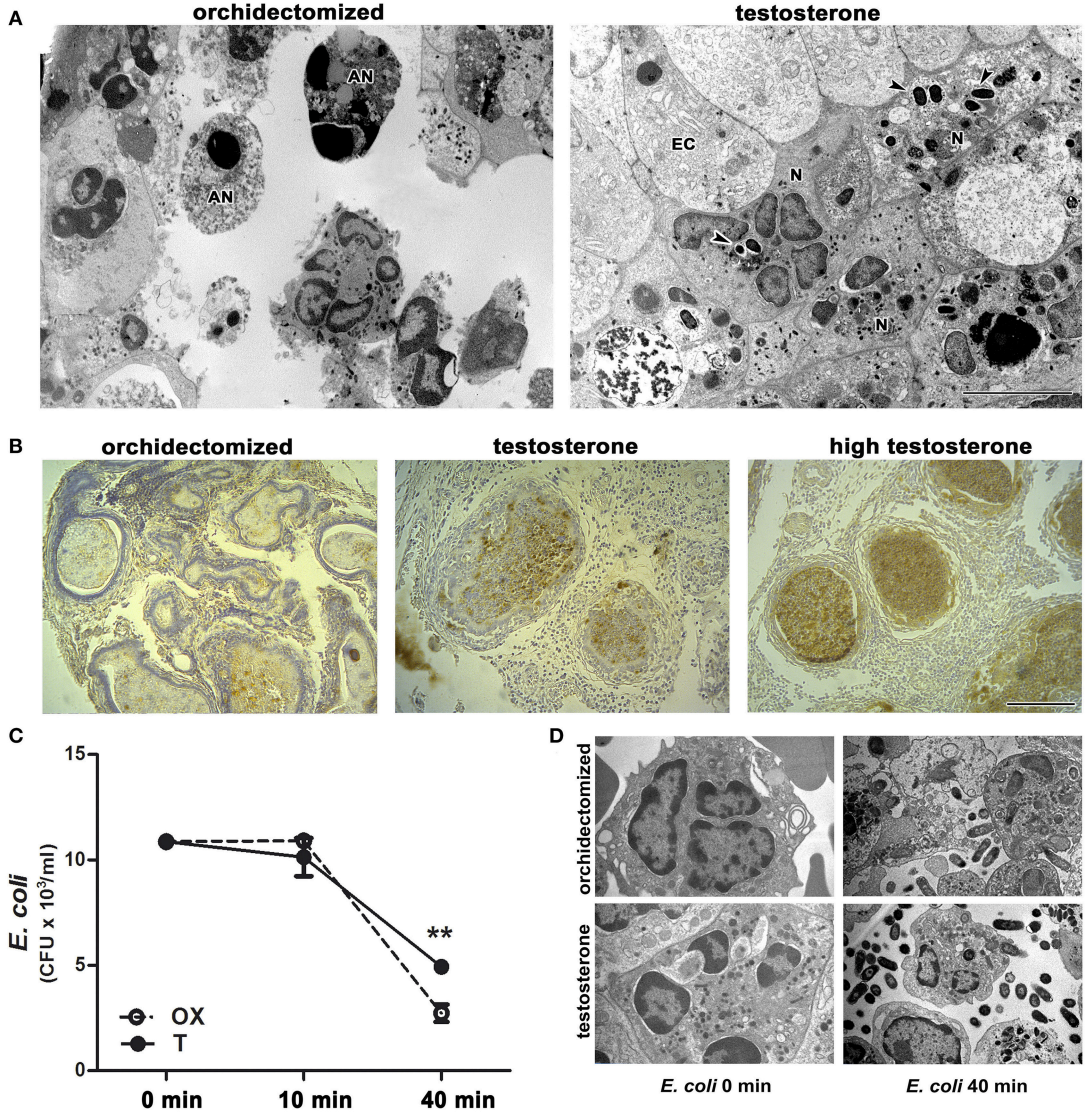
Testosterone treatment impairs neutrophil ability to kill bacteria. **(A-B)** Rats were orchidectomized and treated with testosterone at 2 mg/kg/day or 10 mg/kg/day (high testosterone) before being inoculated with *E. coli* intraprostatically. **(A)** Representative electron microscopy images showing apoptotic neutrophils (AN) free of bacteria (left), while prostate infiltrating neutrophils (N) in testosterone-treated rats display intact phagocytosed bacteria (right, arrowheads) after 5 days of infection. EC: epithelial cell. Bar = 5 μm. **(B)** This is consistent with the intense *E. coli* immunostaining, localized in intracinar neutrophils in testosterone- and high testosterone-treated rats. Bar = 100 μm. **(C–D)** Bacterial growth after being co-incubated *ex vivo* with peritoneal neutrophils from orchidectomized (OX) and testosterone-treated (T) rats. The reduced bactericidal ability of neutrophils from T rats depicting in (**C)** is also seen at ultrastructural level **(D)** where abundant intact free bacteria are observed in presence of testosterone. Data are representative from at least 3 independent experiments. **p < 0.01.

Ultrastructural analysis of the prostate revealed frequent undigested bacteria within neutrophils and invading the epithelial layer in animals treated with testosterone after 5 days of bacterial infection (Figure 4A). This was consistent with an intense immunostaining for *E. coli* in prostates from testosterone-treated rats compared to those from castrated animals (Figure 4B); this was also confirmed by bacterial cultures (data not shown), indicating an abnormal clearance of bacteria in presence of androgens. In contrast, most of neutrophils from castrated animals exhibited apoptotic features and absence of phagocytosed bacteria (Figure 4A), along with a weak *E. coli* immunostaining, all signs compatible with an accurate resolution phase of inflammation. In addition, thioglycollate-elicited peritoneal neutrophils from castrated or testosterone-treated rats were challenged *ex vivo* to *E. coli* and bacterial counting and electron microscopy was performed at 10 and 40 min after coincubation. As shown in Figure 4C, neutrophils from testosterone-treated rats decreased their bactericidal ability. Moreover, the ultrastructural analysis of these cells revealed frequent undigested live bacteria in both the extracellular and intracellular compartments (Figure 4D), confirming that androgens regulate negatively neutrophil activity even in androgen-independent sites.

**Figure 5:**
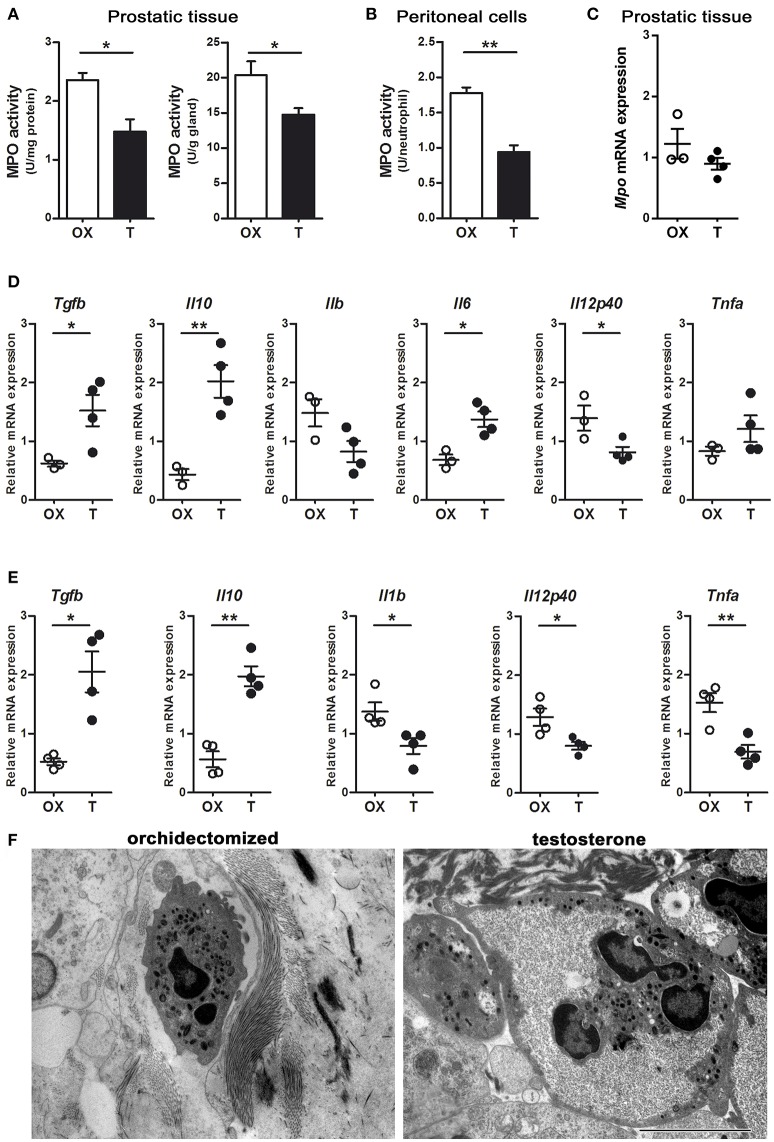
Androgens modulate neutrophil phenotype. Rats were orchidectomized (OX) and treated with testosterone 2 mg/kg/day (T) before being inoculated with 1 mg of LPS intraprostatically for 24 h **(A, C, D, F)**. To elicit peritoneal neutrophils, a single i.p. injection of LPS 1 mg/kg was applied **(B, E)**. **(A)** Myeloperoxidase (MPO) activity in prostatic tissue is impaired in testosterone-treated animals, referred per mg of proteins (left) as well as per g of tissue (right). **(B)** Peritoneal neutrophils also show a decrease in MPO activity in animals treated with T. Data are mean ± SEM, from *n* = 4 per group. *p < 0.05; **p < 0.01. **(C)** The mRNA expression for MPO show no changes between groups. **(D)** Cytokine profiling of Gr (+)-sorted prostatic neutrophils by qPCR, depicting that cells from testosterone-treated animals express high levels of anti-inflammatory TGFβ and IL10, while pro-inflammatory cytokines are reduced. **(E)** Peritoneal neutrophils from testosterone-treated rats also have an anti-inflammatory/ immunomodulatory/“N2-like” phenotype, compatible to that reported by Fridlender et al. (9) and characterized by high expression of TGFβ and IL10 along with low levels of IL1b, IL12p40, and TNFα. ACTB was used as reference mRNA. Mean ± SEM, each dot represents one animal. *p < 0.05; **p < 0.01. **(F)** Representative images of prostatic neutrophils showing cellular edema and vacuolization in testosterone-treated animals. Bar = 5 μm.

MPO is packaged in the azurophilic granules of neutrophils and released into phagosomes when they uptake and kill bacteria (1). We wondered whether the decreased bactericidal effect of neutrophils associated to testosterone could be related to an alteration in the activity of MPO. Prostatic tissues from rats with LPS-induced prostatitis and low level of testosterone showed a higher MPO activity compared to those with normal androgen status (Figure 5A). Consistently, a lower MPO activity per neutrophil was observed in LPS-induced peritoneal neutrophils of animals treated with testosterone (Figure 5B). Interestingly, when the MPO mRNA expression was assessed in Gr (+)-sorted prostate infiltrating neutrophils, no differences were found between groups (Figure 5C), suggesting that androgens regulate MPO at post-transcriptional level.

### Testosterone favors a “N2-like” neutrophil phenotype, with high expression of anti-inflammatory cytokines

Considering the existence of different neutrophil phenotypes, particularly in the tumor microenvironment (9), we wondered if testosterone manipulation could result in a shift of cytokine expression by neutrophils in the context of acute inflammation. By using the LPS-induced prostatitis model, performed in castrated or testosterone-treated rats, the mRNA expression for “N1-” and “N2-like” neutrophils was assessed by qPCR in Gr (+)-sorted cells. Prostatic neutrophils from animals supplemented with testosterone displayed a higher expression of IL10 and TGFβ1 along with a lower IL12 expression compared to those with low testosterone (Figure 5D). When analyzing LPS-recruited peritoneal neutrophils, similar findings were observed, with high testosterone levels being related to high IL10 and TGFβ expressions and to a decrease in the pro-inflammatory cytokines IL12, IL1β, and TNFα (Figure 5E). On the other hand, the morphological evaluation at ultrastructural level showed the existence of clear alterations in LPS-recruited prostatic neutrophils from testosterone-treated animals, including cellular swelling and the occurrence of vacuoles (Figure 5F). These findings suggest that in acute inflammatory scenarios, androgens promote “N2-like” anti-inflammatory and dysfunctional neutrophils, which extend the inflammatory process.

**Figure 6:**
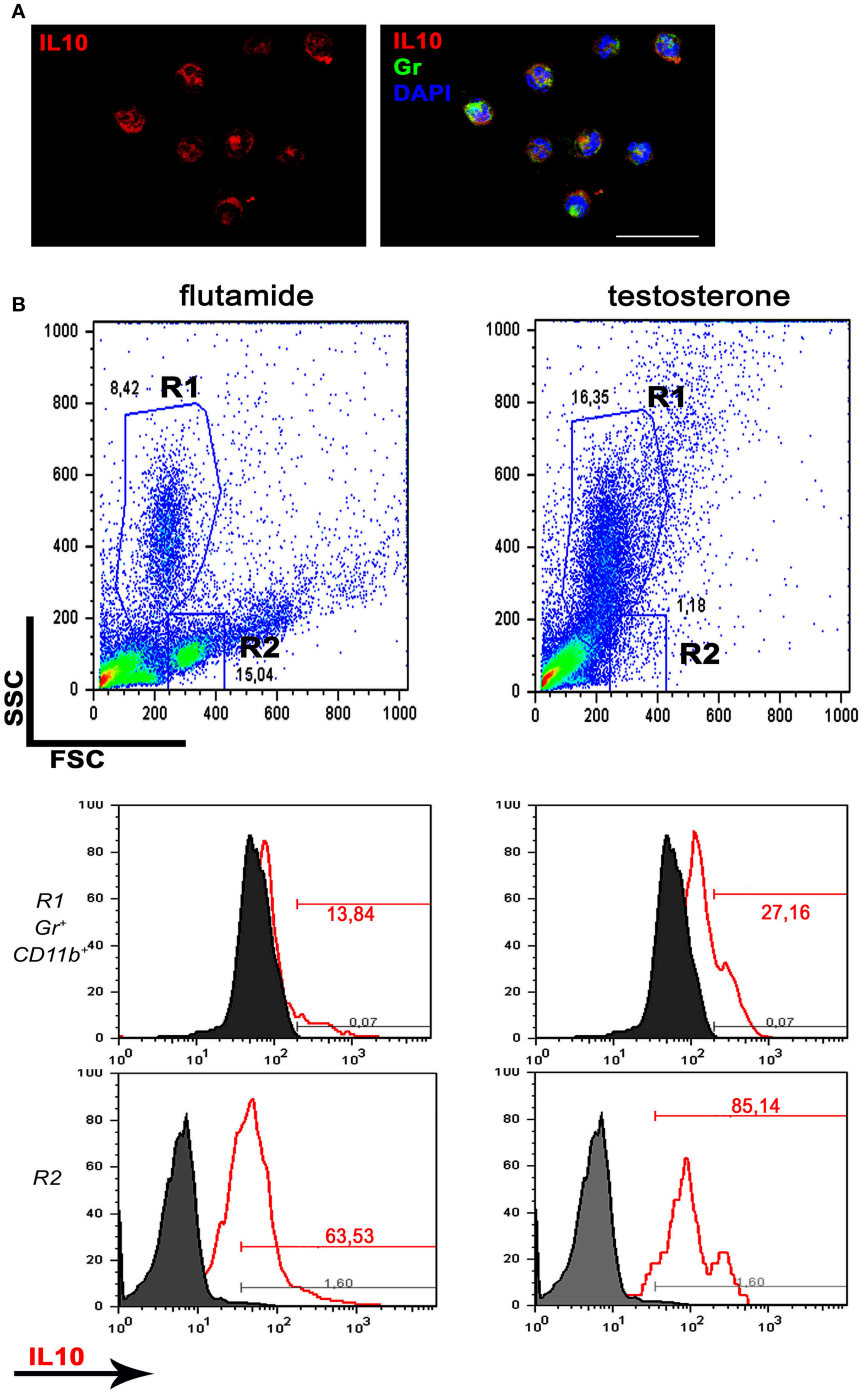
Antiandrogen therapy inhibits IL10 production. Rats were treated with flutamide (7.5 mg/kg/day) for 5 days and thioglycollate-elicited peritoneal cells were pulsed *ex vivo* with LPS 1 μg/ml for 24 h. **(A)** Immunofluorescence for IL10 in absence of antiandrogens, analyzed by confocal microscopy. Most of Gr (+) peritoneal neutrophils express IL10. Bar = 50 μm. **(B)** Flow cytometry showing a decrease in the granulocytic population (R1) along with an increase in the frequency of mononuclear cells (R2) after flutamide treatment (representative dot plots; *n* = 3; top panels). The Gr (+) CD11b (+) R1 neutrophil population shows a decrease in IL10 expression after flutamide treatment (center panels, left) while the monocytic R2 population also displays low expression of IL10 in the same group (bottom panels). Data are representative of *n* = 3.

Together, these data reveal a stimulatory effect of testosterone on neutrophil-produced anti-inflammatory cytokines. To further corroborate these results, rats were treated with flutamide (an antiandrogen widely used in therapy) for 5 days; peritoneal cells were harvested after 4 h of thioglycollate and pulsed *ex vivo* with LPS for 24 h to elicit cytokine secretion. As expected, flow cytometry analysis demonstrated that flutamide decreased the expression of IL10, not only in the granulocytic population but also in mononuclear cells (whose frequency was increased by the anti-androgen, as shown in Figure 6).

## Discussion

Sex differences in mortality and immunocompetence are well documented in humans and other vertebrates (29). Males are at higher risk of developing acute respiratory distress, sepsis, and multiorgan failure after traumatic hemorrhagic shock and thermal injury, in part due to abnormal activation of neutrophils (5). Although morphological differences in granulocytes from men and women have been known since 1954 (30), the need to expose factors explaining functional differences has resurfaced recently in order to adapt new treatments depending on the characteristics of the pathophysiological process in each sex. In this sense, various studies have uncovered important immune regulatory functions for androgens, including effects on neutrophil accumulation (31–33), maturation, activation (34), and survival (35). In line with this evidence, we here report that testosterone increases local chemokine expression, leading to a higher recruitment of neutrophils to the site of infection, but at the same time, these cells exhibit a “N2-like” phenotype with a reduced efficiency in killing bacteria and high expression of immunomodulatory molecules such as IL10 and TGFβ1.

Neutrophil recruitment is an important early step in inflammatory response against pathogenic invasion or sterile tissue damage. The recruitment of neutrophils into the tissue is initiated by neutrophil-active chemoattractants, mainly CXCR2 ligands CXCL1 and CXCL2, released from danger signal-activated professional tissue-resident sentinel cells or stromal cells (28). Recruited neutrophils into injury site can also deliver active chemokines directly contributing to their own recruitment (36). In the present study, we observed that the presence of testosterone leads to an increased bacterial-induced mRNA expression of CXCL1 and CXCL2, which was associated to a higher neutrophil recruitment. In agreement, androgen supplementation has been shown to augment neutrophil infiltration in penile urethroplasty (33) and after myocardial infarction in both male and females (31). Human studies demonstrated that decreasing testosterone levels results in an attenuation of exercise-induced neutrophil accumulation in muscles (37), indicating a strong positive correlation between androgen levels and neutrophil infiltration. Furthermore, hyperandrogenemia is associated to a higher neutrophil count in steady state conditions in women with polycystic ovary syndrome (38). The excessive or aberrant neutrophil infiltration in the presence of high androgen levels seems to favor tissue damage and organ dysfunction not only after infection but also in non-infectious conditions (31, 33). However, novel evidence suggests that neutrophil accumulation could also play a positive role in organ homeostasis by resolving inflammation (39–42), with a phenotypic characterization (i.e., pro-inflammatory vs. pro-resolving neutrophils) being necessary in order to predict the final effect of these cells on damage progression and tissue function.

Unlike other immune cells, existence of clearly defined neutrophils subtypes remains unclear. Accumulating evidence suggests that neutrophils may exhibit certain plasticity according to the microenvironment (8, 9, 43, 44). For instance, different subtypes of neutrophils were identified during infection with methicillin-resistant *S. aureus* (MRSA), associated either to resistance or to susceptibility to infection in mice (8). Neutrophils from MRSA-resistant hosts show a pro-inflammatory phenotype, with IL12 and CCL3 production, whereas those from MRSA- susceptible mice are anti-inflammatory in nature (IL10+/CCL2+), inducing M2 macrophages (8). Neutrophil polarization to an IL10-producing anti-inflammatory phenotype has also been reported by different pathogens (43, 45) as well as by serum amyloid A1 (46). Nevertheless, neutrophil subsets have mainly been characterized in tumoral conditions, where constitutively produced cytokines and growth factors can promote polarization of cells recruited into the tumor (9, 47). Of note, IFNβ and TGFβ1 induce neutrophils to acquire anti-tumoral (N1) or pro-tumoral (N2) phenotypes respectively (9, 47). N2 neutrophils are postulated to have a main role in promoting tumoral growth by increasing extracellular matrix deposition and by dampening a proper immune response (9). In this context, TGFβ1 appears as a central player in the tumor microenvironment orchestrating diverse pro-tumoral, anti-inflammatory, and immunomodulatory actions, including the induction and maintenance of an N2 phenotype. The induction of “N2-like” neutrophils in the presence of testosterone in our study can also be explained by overexpression of TGFβ1 since its promoter activity is directly regulated by androgens through the androgen receptor (AR) (48, 49). However, whether TGFβ1 acts directly or by other mechanisms to induce N2 neutrophil maturation by androgens deserves further research.

The ability of androgens to promote anti-inflammatory/immunomodulatory phenotypes has been previously recognized for professional immune cells (16, 20). In general, testosterone tends to inhibit pro-inflammatory molecules such as TNFα, iNOS, and NO whereas induces IL10 and TGFβ1 anti-inflammatory signaling (19, 50–52). Accordingly, we found that androgens favor a higher IL10 and TGFβ1 expression along with a lower IL12 expression on recruited neutrophils. Strikingly, this phenotype was accompanied by a reduction in both MPO activity and bactericidal ability *in vivo* as well as *ex vivo*, resulting in an inadequate bacterial clearance. In accordance, it has been reported that, *in vitro*, testosterone decreases the microbicidal activity of human neutrophils by dampening the production of reactive oxygen species (53, 54). In contrast, after trauma or burn injury, androgens have been reported to enhance neutrophil activation in blood, as judged by CD11b expression and respiratory burst activity (32), which might explain why males are more susceptible to acute shock aggressiveness than females. These ambivalent results could be attributed to the type of stimuli used and the pathological conditions, with scarce data available on the bactericidal ability of neutrophils after androgen manipulation. In any case, the promotion of “N2-like” neutrophils by androgens may be deleterious as well as favorable, depending on the particular context; albeit being inefficient in killing bacteria, their ability to generate TGFβ1, as described before (39, 40), could be beneficial in repairing tissues and resolving inflammation (39, 40).

Our results indicate that testosterone modulates neutrophil activity within the prostate and in androgen-independent sites as well. In a bacterial milieu, testosterone promotes, in a dose dependent manner, a recruitment of malfunctioning neutrophil that amplifies and prolongs the inflammatory response, with the persistence of their toxic products destroying cellular components and generating a favorable environment for the development of pathologies (14, 55). On the other hand, the testosterone-induced anti-inflammatory profile displayed by these neutrophils could be beneficial in some non-bacterial types of inflammation.

## Author contributions

MS and AQ performed most protocols. MS, NP, MC, CL, and AQ analyzed data. JN carried out and analyzed qPCR experiments. GM performed, analyzed, and supervised intravital imaging. MS, CM, and AQ wrote the manuscript. CM and AQ designed the study and supervised the research work.

### Conflict of interest statement

The authors declare that the research was conducted in the absence of any commercial or financial relationships that could be construed as a potential conflict of interest. The reviewer PM and handling Editor declared their shared affiliation.

## Supplementary material

Supplementary Movie 1 Testosterone increases LPS-induced neutrophil accumulation in the liver. Intravital microscopy after an i.p. LPS 0.5 mg/kg injection for the visualization of neutrophil recruitment to liver sinusoids. Mice were previously treated with the antiandrogen flutamide (7 mg/kg/day) or with testosterone (10 mg/kg/day) for 3 days. Quantification of Ly6G (+) neutrophils (green) is shown in Figure 2D.

Supplementary Table 1 List of primers *(Rattus norvegicus)* used for different target mRNAs.
