# Supplementary material for: Inefficient N2-Like Neutrophils Are Promoted by Androgens During Infection
Source: Front Immunol. 2018 Sep 3;9:1980. doi: 10.3389/fimmu.2018.01980 (PMC6129603; doi:10.3389/fimmu.2018.01980)
Supplement: Supplementary Table 1 — List of primers (Rattus norvegicus) used for different target mRNAs. [file Table_1.DOCX]

| Name | Sense (5'→3') | Antisense (5'→3') |
| --- | --- | --- |
| *Actb* | GGCACCACACTTTCTACAATG | TGGCTGGGGTGTTGAAGGT |
| *Tnfa* | CCACCACGCTCTTCTGTC | CTACGGGCTTGTCACTCG |
| *Tgfb* | TGAACCAAGGAGACGGAATACAGG | GCCATGAGGAGCAGGAAGGG |
| *Il1b* | CTTCGAGATGAACAACAA | CATGGAGAATACCACTTG |
| *Il6* | CAGAGTCATTCAGAGCAATAC | GATGGTCTTGGTCCTTAGCC |
| *Il10* | GAAGCTGAAGACCCTCTGGA | CGGGTGGTTCAATTTTTCAT |
| *Il12p40* | TGGAGCACTCCCCATTCCTA | ACGAGGAACGCACCTTTCTG |
| *Cxcl1* | GCGGAGAGATGAGAGTCTGG | GAGACGAGAAGGAGCATTGG |
| *Cxcl2* | AGATATGCATTGGCCAGCTC | TCACAGTGTGGAGGTGGTGT |
| *Mcp1* | TCTTCCTCCACCACTATGC | TCTCCAGCCGACTCATTG |
| *Mpo* | CGATAGGTTTTGGTGGGAGA | TCCAGGAAGCCAGATTCAGT |

**Supplementary table 1.** List of primers (*Rattus norvegicus*) used for different target mRNAs.
